# Supplementary material for: Smooth Interpolating Curves with Local Control and Monotone Alternating Curvature
Source: Comput Graph Forum. 2022 Oct 6;41(5):25–38. doi: 10.1111/cgf.14600 (PMC9827861; doi:10.1111/cgf.14600)
Supplement: Supplementary file 1 — Supplement Material [file CGF-41-25-s001.zip › Local-Smooth-Interpolating-MonoCurvature/extern/clothoids/submodules/Utils/ThirdParties/fmt/fmt-8.0.1.zip!/fmt-8.0.1/doc/html/genindex.html]

Index — fmt 8.0.1 documentation


Toggle navigation

{fmt}

- 8.0.1
  - 8.0.1
  - 8.0.0
  - 7.1.3
- Contents
- Usage
- API
- Syntax

# Index

**F**

## F

|  |  |
| --- | --- |
| - fmt::arg (C++ function) - fmt::basic\_format\_arg (C++ class) - fmt::basic\_format\_args (C++ class) - fmt::basic\_format\_args::basic\_format\_args (C++ function), [1], [2] - fmt::basic\_format\_args::get (C++ function) - fmt::basic\_format\_context (C++ class) - fmt::basic\_format\_context::basic\_format\_context (C++ function) - fmt::basic\_format\_context::char\_type (C++ type) - fmt::basic\_format\_parse\_context (C++ class) - fmt::basic\_format\_parse\_context::advance\_to (C++ function) - fmt::basic\_format\_parse\_context::begin (C++ function) - fmt::basic\_format\_parse\_context::check\_arg\_id (C++ function) - fmt::basic\_format\_parse\_context::end (C++ function) - fmt::basic\_format\_parse\_context::next\_arg\_id (C++ function) - fmt::basic\_memory\_buffer (C++ class) - fmt::basic\_memory\_buffer::basic\_memory\_buffer (C++ function) - fmt::basic\_memory\_buffer::grow (C++ function) - fmt::basic\_memory\_buffer::operator= (C++ function) - fmt::basic\_memory\_buffer::reserve (C++ function) - fmt::basic\_memory\_buffer::resize (C++ function) - fmt::basic\_string\_view (C++ class) - fmt::basic\_string\_view::basic\_string\_view (C++ function), [1], [2] - fmt::basic\_string\_view::data (C++ function) - fmt::basic\_string\_view::size (C++ function) - fmt::bg (C++ function) - fmt::buffered\_file::vprint (C++ function) - fmt::detail::buffer (C++ class) - fmt::detail::buffer::append (C++ function) - fmt::detail::buffer::capacity (C++ function) - fmt::detail::buffer::clear (C++ function) - fmt::detail::buffer::data (C++ function), [1] - fmt::detail::buffer::size (C++ function) - fmt::dynamic\_format\_arg\_store (C++ class) - fmt::fg (C++ function) - fmt::format (C++ function) - fmt::format\_arg\_store (C++ class) | - fmt::format\_args (C++ type) - fmt::format\_context (C++ type) - fmt::format\_system\_error (C++ function) - fmt::format\_to (C++ function) - fmt::format\_to\_n (C++ function) - fmt::format\_to\_n\_result (C++ struct) - fmt::format\_to\_n\_result::out (C++ member) - fmt::format\_to\_n\_result::size (C++ member) - fmt::formatted\_size (C++ function) - fmt::fprintf (C++ function) - fmt::gmtime (C++ function) - fmt::is\_char (C++ struct) - fmt::join (C++ function), [1] - fmt::localtime (C++ function) - fmt::make\_args\_checked (C++ function) - fmt::make\_format\_args (C++ function) - fmt::operator""\_a (C++ function) - fmt::operator""\_format (C++ function) - fmt::ostream (C++ class) - fmt::ostream::output\_file (C++ function) - fmt::ostream::print (C++ function) - fmt::print (C++ function), [1], [2], [3] - fmt::printf (C++ function) - fmt::ptr (C++ function), [1], [2] - fmt::sprintf (C++ function) - fmt::string\_view (C++ type) - fmt::system\_error (C++ function) - fmt::to\_string (C++ function) - fmt::to\_string\_view (C++ function) - fmt::to\_wstring (C++ function) - fmt::vformat (C++ function) - fmt::vprint (C++ function) - fmt::wformat\_context (C++ type) - fmt::wstring\_view (C++ type) - FMT\_COMPILE (C macro) - FMT\_STRING (C macro) |

© Copyright 2012-present, Victor Zverovich.
Created using Sphinx 3.3.0.
